# Structural variations of Si_1−*x*_C_*x*_ and their light absorption controllability

**DOI:** 10.1186/1556-276X-7-503

**Published:** 2012-09-06

**Authors:** Jihyun Moon, Seung Jae Baik, Byungsung O, Jeong Chul Lee

**Affiliations:** 1 KIER-UNIST Advanced Center for Energy, Korea Institute of Energy Research, 152 Gajeong-ro, Yuseong-gu, Daejeon, 305-343, South Korea; 2 Department of Physics, Chungnam National University, Yuseong-gu, Daejeon, 305-764, South Korea; 3 Department of Electrical, Electronic, and Control Engineering, Hankyong National University, Anseong-si, Gyeonggi-do, 456-749, South Korea

**Keywords:** Nanocrystalline Si, Solar cell, Silicon carbide, Light absorption, Superlattice, PACS, 78.20. + e, 78.30.Ly, 78.40.Fy.

## Abstract

The emergence of third-generation photovoltaics based on Si relies on tunable bandgap materials with embedded nanocrystalline Si. One of the most promising approaches is based on the mixed-phase Si_1 − *x*_C_*x*_. We have investigated the light absorption controllability of nanocrystalline Si-embedded Si_1 − *x*_C_*x*_ produced by thermal annealing of the Si-rich Si_1 − *x*_C_*x*_ and composition-modulated superlattice structure. In addition, stoichiometric SiC was also investigated to comparatively analyze the characteristic differences. As a result, it was found that stoichiometric changes of the matrix material and incorporation of oxygen play key roles in light absorption controllability. Based on the results of this work and literature, a design strategy of nanocrystalline Si-embedded absorber materials for third-generation photovoltaics is discussed.

## Background

Amorphous materials with embedded nanocrystals enable a design method for specific optical and electrical properties of thin film materials. This design enablement of this mixed-phase material originates from the well-known physical principle called quantum confinement. The size-dependent bandgap tuning of nanocrystals embedded in a material with a larger bandgap has been experimentally demonstrated by several groups [1-3], and its application has been also successfully demonstrated in the fields of single-electron devices [4], memories [5], light-emitting devices [6], and solar cells [7]. In solar cells, nanocrystals and their quantum confinement serve a route to the third-generation photovoltaics [8]. For example, intermediate band solar cells [9] and multi-exciton collection [7,10] have been demonstrated, which were expected to provide groundbreaking enhancement of solar cell efficiency. However, those demonstrations for third-generation photovoltaics are based on III-V epitaxial thin films or lead chalcogenide-based colloidal nanocrystals, which might not be cost-effective or environmentally friendly. On the other hand, an aggressive consideration called all-Si tandem solar cells is under research in some research groups [11]. They have suggested multi-junction solar cells composed of silicon nanocrystals whose bandgaps are controlled by their sizes. Some fundamental works such as size-dependent photoluminescence wavelength [1,2,11], window layer application of heterojunction Si solar cells [12], and primitive absorber layer application in thin film Si solar cells [13] have been reported.

**Figure 1:**
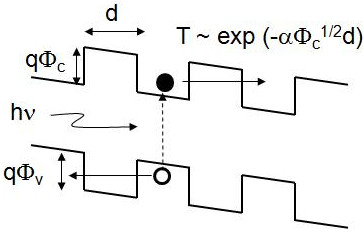
**Band diagram of a nanocrystalline material embedded in a matrix material with larger bandgap.** Conduction band discontinuity (*qΦ*_c_), valence band discontinuity (*qΦ*_v_), and inter-nanocrystal distance (*d*) are indicated. Electron-hole pair generation with incident light (hν) and approximate expression of nanocrystal-to-nanocrystal tunneling probability (*T*) are also shown.

According to the operation principle of solar cells, the light absorber should provide efficient carrier separation as well as generation of electron-hole pairs upon light irradiation. That is, not only the optical absorption property, but also the electrical conductivity of the absorber material is essential for solar cell application. In a mixed-phase material, the band discontinuity *Φ*_c_ (similar for the valence band discontinuity) at the nanocrystalline Si/matrix interface and the inter-nanocrystal distance *d* indicated in Figure 1 determine the low-field conductivity of the material when *Φ*_c_ is much larger than the thermal energy kT (approximately 26 meV at room temperature). Therefore, matrix materials with large band discontinuities with crystalline Si such as SiO_*x*_[1] or SiN_*x*_[2] would not seem to provide appropriate electrical properties for solar cell applications. In this regard, among the candidate methods to implement Si nanocrystals as a light absorber in solar cells, SiC-based materials, i.e., Si nanocrystals embedded in Si_1 − *x*_C_*x*_[14-16], would be most promising.

Previous works on the Si nanocrystal in Si_1 − *x*_C_*x*_ demonstrated that thermal annealing can be used to control the bandgap of this mixed-phase film within the range between 1.4 and 2.2 eV, which renders the optimal combination of triple-junction all-Si tandem solar cells. This bandgap variation was mainly attributed to the bandgap increase of the Si_1 − *x*_C_*x*_ matrix due to the limited effect of the quantum confinement of Si nanocrystals [14]. According to this argument, the Si nanocrystal is not necessary in the formation of all-Si tandem solar cells, which is well supported by earlier findings on the bandgap tunability of amorphous SiC [17]. Nevertheless, the inclusion of silicon nanocrystal would provide additional opportunities for breaking the Shockley-Queisser limit [18] via intermediate band solar cells [9] or multiple exciton generation [19]. Therefore, the research on the Si nanocrystal is still meaningful for the potential third-generation photovoltaics. In this work, we have performed structural and optical characterization of thermally annealed SiC thin films with structural variations. As a result of systematic analysis, we have found that nanocrystalline Si (nc-Si) formation significantly affects the optical properties due to the stoichiometric changes of the matrix material, which also seems to be related to the oxygen incorporation. This coupled effect of stoichiometric change and oxygen incorporation will be discussed in detail, and a novel strategy on the tunable absorber design of solar cells will be also presented.

## Methods

Si_1 − *x*_C_*x*_ thin films were deposited on Si wafers and quartz substrates simultaneously by radio frequency (RF) magnetron co-sputtering at 200°C. High-purity (4N) Si and C targets (diameter, 4 in.) were used. After cleaning the substrates, the Si wafers were dipped in 5% HF solution for 1 min just before loading into the chamber to remove native oxides. The composition of Si_1 − *x*_C_*x*_ films was controlled by adjusting RF powers to each target material. We have chosen two kinds of composition for the annealing experiment of Si_1 − *x*_C_*x*_: stoichiometric SiC (SSC) with *x* = 0.56 and Si-rich SiC (SRSC) with *x* = 0.08, where the composition was characterized by Rutherford backscattering spectroscopy. SSC and SRSC samples were prepared to have a film thickness of 150 nm for all the experiment and characterization. In addition, superlattice structures (SL) were also prepared by alternative deposition of 36 periods of SSC layers (approximately 1 nm) and SRSC layers (approximately 4 nm), which has a total film thickness around 180 nm. Thermal annealing experiments for SSC, SRSC, and SL samples were performed in a quartz tube furnace at 800°C, 900°C, and 1,000°C for 20 min in nitrogen atmosphere.

The structural and crystallographic characterization of the nanocrystals in the SL was performed by high-resolution transmission electron microscopy (HRTEM), transmission electron diffraction (TED), and grazing incidence X-ray diffraction (GIXRD). Raman spectroscopy was used to analyze the crystal volume fractions, and chemical bonding configurations were studied with Fourier transform infrared (FTIR) spectroscopy and X-ray photoemission spectroscopy (XPS). Photoluminescence (PL) characteristics were studied with an Ar^+^ laser (*λ* = 488 nm) excitation source within the temperature range from 5 K to room temperature. Optical transmission and reflection measurements within the wavelength range between 300 and 1,800 nm were performed with an ultraviolet-visible-near infrared spectrophotometer, and optical bandgaps were determined from the Tauc plot.

## Results and discussion

**Figure 2:**
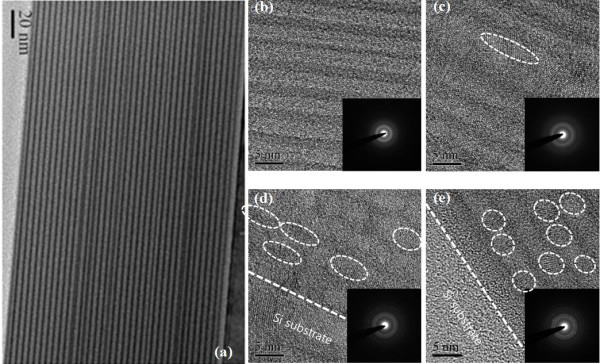
**TEM and TED images.** (**a**) TEM image of the as-deposited SSC/SRSC superlattice. HRTEM and TED images of SL: (**b**) as-deposited and annealed at (**c**) 800°C, (**d**) 900°C, and (**e**) 1,000°C. Dashed lines indicate the surface of substrate Si wafer, and dashed circles indicate the boundaries of nanocrystalline Si.

To confirm the thickness and nanocrystal formation upon annealing, TEM analysis was performed using the SL samples with various annealing conditions. Figure 2a shows the as-deposited SL, where dark layers represent SSC and bright layers represent SRSC [15]. Thirty-six periods are clearly observed, and the thicknesses of SSC and SRSC layers were found to be 1 nm and 4 nm as expected. Figure 2b,c,d,e shows the HRTEM and TED images of the as-deposited SL and SLs annealed at 800°C, 900°C, and 1,000°C. There is no indication of nc-Si in the image of the as-deposited sample, while we can find some lattice fringes of nc-Si from the images of the annealed samples. Areal densities and average lateral sizes of nc-Si within SRSC layers were estimated by observing TEM images from several different locations in the sample. As the annealing temperature increases, the density of nc-Si increases, while the average sizes of nc-Si decrease. The estimated areal density and the average sizes are as follows: 2.4 × 10^8^ cm^−2^, 16 nm in the 800°C annealed sample; 2.9 × 10^10^ cm^−2^, 8 nm in the 900°C annealed sample; and 3.8 × 10^11^ cm^−2^, 5 nm in the 1,000°C annealed sample. The heights of the nanocrystallites were all less than 4 nm, which is the thickness of the SRSC layers. Accordingly, the shapes of the nanocrystallites were ellipsoids with a long axis on the horizontal direction with an annealing temperature of 800°C, and it becomes more spherical; the lateral size is decreased as the annealing temperature is increased.

**Figure 3:**
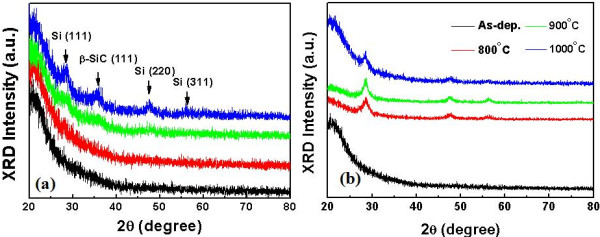
**GIXRD spectra.** (**a**) GIXRD spectra of SL: as-deposited and annealed at 800°C, 900°C, and 1,000°C. (**b**) GIXRD spectra of SRSC: as-deposited and annealed at 800°C, 900°C, and 1,000°C.

While thermal annealing induces the growth of nanocrystallites in SiO_*x*_ or SiN_*x*_[1,2], the average size of the nanocrystallites in SiC_*x*_ decreases as the annealing temperature increases. This can be understood by the increase of Si-C bonds and breakage of Si-Si bonds as the annealing temperature increases, which was also pointed out previously [14]. Moreover, it was reported that when *x* in SiC_*x*_ is smaller than 0.2, only Si nanocrystals are observed; however, when *x* is larger than 0.2, both Si and SiC crystals are observed [16]. GIXRD spectra shown in Figure 3 confirm the annealing temperature-dependent nanocrystal formation. Crystal peaks are more pronounced for SRSC than for SL especially at low annealing temperatures, which are due to the larger probability of Si-Si bond formation for SRSC compared to SL. The formation of β-SiC is clearly shown in Figure 3a; however, β-SiC is not formed in SRSC (see Figure 3b) and SSC (not shown) even at the annealing temperature of 1,000°C. The case of SRSC is consistent with the above-mentioned prior work [16], and this may be due to the limited supply of carbon atoms for the growth of β-SiC nanocrystallites. For the case of SSC, considering that the crystallization temperature of SiC is higher than 1,000°C [19], it is reasonable that the SSC layer remains as an amorphous phase after annealing at 1,000°C. This observation states that the activation energy of β-SiC formation in amorphous SiC may be reduced with a lot of Si-Si bond-forming reactions.

**Figure 4:**
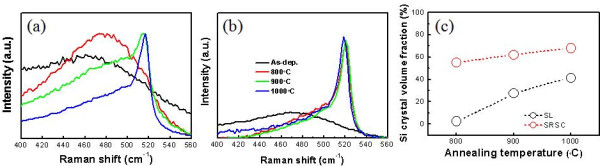
**Raman spectra and volume fractions.** Raman spectra of (**a**) SL and (**b**) SRSC for as-deposited and annealed samples at 800°C, 900°C, and 1,000°C. (**c**) nc-Si volume fractions of each samples extracted from the Raman spectra.

The volume fraction of nc-Si is determined from the Raman spectra shown in Figure 4. The crystalline volume fraction of the annealed SRSC is far higher than that of SL, which is partly related to the amount of excess Si atoms within the thin film: it can be estimated from the composition analysis of each thin film that the elemental fraction of excess Si for SRSC is 84% and that of excess Si for SL is 64.8%. Considering the difference in annealing temperature dependence of the crystalline volume fraction for SRSC and SL, it seems that the effect of interface energy contributes to the retarded crystallization in SL [20]. In addition, the peak shift towards a low wavenumber compared to the bulk Si value (520 cm^−1^) for SL can be attributed to the effect of strain in Si nanocrystallites [15,21], while the peak shifts are not prominent for SRSC films annealed below 1,000°C. In SL, spacer SSC may provide strain in nc-Si because the spacer SSC restrains the vertical growth of nc-Si. In SRSC, the growth direction of nc-Si is randomly allowed, and individual crystal growth may happen along the direction with minimum strain on each nc-Si.

**Figure 5:**
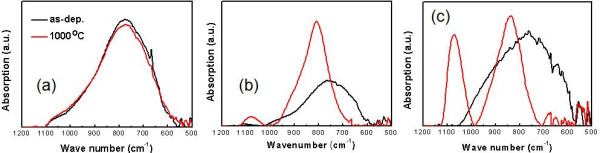
FTIR spectra of (a) SSC, (b) SL, and (c) SRSC for as-deposited and 1,000°C annealed samples.

In the FTIR absorption spectra shown in Figure 5, two absorption modes are dominantly observed. The peak near 760 cm^−1^ for the as-deposited films represent the Si-C stretching vibration mode, and it shifts towards a higher wavenumber after annealing for SRSC and SL [15], while the peak shift was not observed after annealing for SSC. The peak shifts after annealing for SRSC and SL are similar to previous reports [14,15], which were attributed to the increased number of Si-C bond and crystalline Si-C bond formation. These results are consistent with the β-SiC formation from the GIXRD spectra shown in Figure 3a for SL, and GIXRD did not resolve the β-SiC formation for SRSC probably due to the suppressed crystal growth of β-SiC caused by low C composition. The second peak near 1,070 cm^−1^ corresponds to the Si-O stretching vibration mode [16]. The increase of this peak implies the thermal oxidation of thin films during the annealing process, when the atmosphere was nitrogen gas with an uncontrolled small-amount incorporation of oxygen. The amount of Si-O bond does not show a significant increase after annealing for SSC, but for SRSC, the Si-O bond density seems to become comparable with the Si-C bond after annealing. This means that SSC can play as a diffusion barrier for oxygen molecules, while in SRSC, not only surface oxidation, but also oxygen diffusion into the film could happen. The enhancement of oxygen diffusion through nc-Si surfaces in highly crystalline mixed-phase Si thin films is already pointed out before [22,23]. The impact of oxygen diffusion in SL is not as pronounced as that in SRSC as shown in Figure 5b. This is because of the periodically inserted SSC layers, which can limit a large amount of oxygen diffusion into the film. Additionally, small peaks shown in 500 to 700 cm^−1^ can be attributed to H_2_O (800 to 600 cm^−1^) and/or CO_2_ (667 cm^−1^) [24].

**Figure 6:**
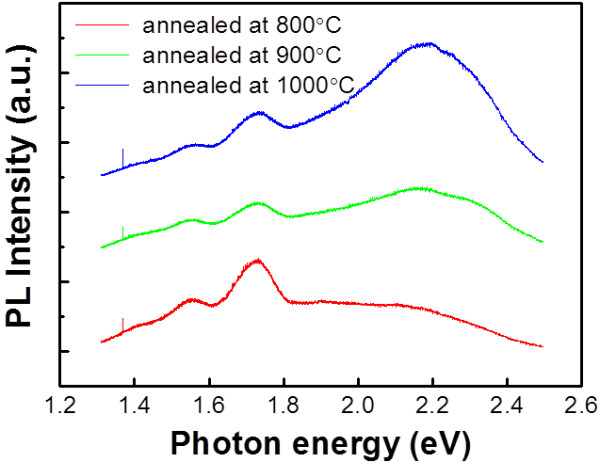
Low-temperature PL spectra from SL annealed at several temperatures.

In Figure 6, the PL spectra of SL with different annealing temperatures are shown (and no PL signal was observed for the as-deposited SL). Three peaks are pronounced at around 1.55 eV, 1.72 eV, and 2.2 eV. The energy range of the lower two peaks coincides with that of the reported emission from nc-Si in SiO_*x*_[1], but the intensity and emission energy do not have any dependence with the crystalline volume fraction, i.e., annealing temperature. Assuming these two peaks originate from nc-Si, the intensity should increase with increasing crystalline volume fraction. In addition, the emission energy should shift towards a higher energy with higher annealing temperature because the higher annealing temperature resulted in smaller nc-Si as shown in Figure 2. Therefore, these two peaks do not seem to be related to nc-Si, but we can attribute them to surface defects of Si nanoclusters [25,26]. In addition, the intensity of the broad peak near 2.2 eV increases as the annealing temperature increases, and the emission peak slightly shifts towards a higher energy. Referring to the above analysis on FTIR spectra, the amount of the incorporated oxygen also increases as the annealing temperature increases. In oxygen-incorporated SiC (SiCO) thin films, it was reported that PL emission from 2 to 3 eV increases and that emission peaks blueshift as the amount of incorporated oxygen increases [27]. Therefore, the 2.2-eV PL peak can be attributed to the SiCO formation due to the small amount of oxygen diffusion as confirmed by the FTIR analysis shown in Figure 5. In addition, the behavior of the 2.2-eV peak does not seem to be related to nc-Si formation because the theoretical bandgap of the 4- to 5-nm-sized nanocrystal is only around 1.6 to 1.8 eV [28].

**Figure 7:**
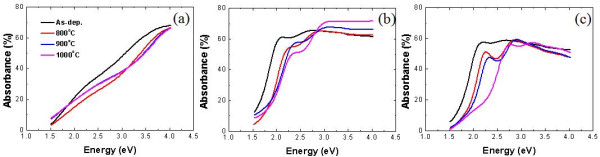
**Optical absorbance spectra evaluated by 100% transmittance and reflectance.** (**a**) SSC, (**b**) SL, and (**c**) SRSC for as-deposited and 800°C, 900°C, and 1,000°C annealed samples.

**Figure 8:**
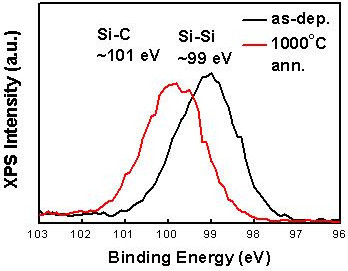
**Si 2*p*XPS spectra of as-deposited and 1,000°Cannealed samples.** Surface layer (approximately 50 nm) is removed before characterization to eliminate the surface oxidation effect (initial sample thickness was 150 nm). After annealing, the Si 2*p* peak is shifted towards a higher energy, which indicates the increment of Si-C bond density.

Thermal annealing induces nc-Si formation and oxygen incorporation in SRSC and SL films, and these lead to variation in optical properties. In Figure 7, the optical absorbance characteristics of each film with various annealing conditions are shown. SSC shows a slight decrease in absorbance as annealing temperature increases. By considering that there are no significant changes in crystallization and bonding configuration, this decrease of absorbance in the low-energy regime is attributed to the increased reflectance at the SSC/air interface due to the thin thermal oxide formation at the surface. In addition, the decrease of absorbance in the high-energy regime can be attributed to the structural changes in the amorphous SiC network, which is supported by the XPS spectra shown in Figure 8. After annealing SSC at 1,000°C, the chemical shift of Si 2*p* spectra implies the increase of the Si-C bond density [14].

**Figure 9:**
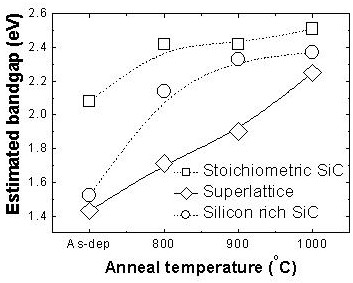
Optical bandgap extracted by Tauc's plot for SSC, SL, and SRSC with various annealing conditions.

The absorbance of SL as shown in Figure 7b shows two distinct features. In the low-energy regime, the absorbance curve blueshifts as the annealing temperature increases (or optical gap increases as shown in Figure 9, and in the high-energy regime, the absorbance increases as the annealing temperature increases. The oxygen incorporation can explain the blueshifts of absorbance with increased annealing temperature, and this similar effect is reproduced and more pronounced in SRSC as shown in Figure 7c. These are consistent with the amount of oxygen incorporation in these films at different annealing temperatures as discussed above with the FTIR spectra. There is another distinct feature of the SL absorbance with different annealing temperatures in the high-energy regime, that is, the increase of the absorbance as the annealing temperature increases, which is not reproduced in the SRSC film. The difference in the volume fractions of SL and SRSC nc-Si does not explain these differences in the absorption spectra, and nc-Si does not seem to play a significant role in optical absorption in the high-energy regime because of its lower absorption coefficient in the high-energy regime compared to amorphous Si or amorphous Si alloys [29,30]. Therefore, these different absorption spectra between SL and SRSC in the high-energy regime may be related to the difference in the matrix material. With thermal annealing at 1,000°C, the effective composition of the matrix material of SRSC is evaluated to be Si_0.75_C_0.25_, and that of SL is evaluated to be Si_0.21_C_0.79_ (these compositions are calculated from the initial compositions and nc-Si volume fractions). The matrix of SRSC is still Si-rich, while that of SL is C-rich. Therefore, these absorbance characteristics in the high-energy regime state that the more C-rich matrix exhibits larger absorption in the high-energy regime. It is known that the larger number of C-C bond diversifies optical properties, which is a strong function of bond configuration as studied in amorphous carbon materials [31]. We speculate that the matrix material with larger composition of C produces more *sp*^2^ bonds than *sp*^3^ bonds in our thermal annealing experiment.

Light absorption in Si_1 − *x*_C_*x*_ is basically controlled by its stoichiometry [17] and bonding configurations [32]. This may enable the all-Si_1 − *x*_*C*_*x*_ tandem solar cell structure, but employing the mixed-phase structure, i.e., nc-Si with various Si_1 − *x*_C_*x*_, would provide more opportunities in high-efficiency strategies such as intermediate bands or multiple exciton generation. It is evident that tuning the sizes of nc-Si is not a very efficient method to cover a broad range of absorption band, but tuning the stoichiometry of the matrix material would be highly viable. In addition, both the stoichiometry of the matrix material and the oxygen incorporation can be applied to tune the absorption property of the material. In this work and several previous reports [14-16], thermal annealing methods have been presented to demonstrate bandgap tuning properties of mixed-phase Si_1 − *x*_C_*x*_ thin films; however, direct forming methods using low-temperature deposition tools are highly necessary to attain progresses towards device demonstration. There have been several reports regarding the mixed-phase Si_1 − *x*_C_*x*_ thin film using low-temperature processes [33,34], polymorphous Si thin films in fast deposition regime [35-37], and formation of nc-Si using atomic hydrogen treatment which have been known to be feasible for photovoltaic thin film production [38]. Using these pre-existing technologies, further investigation on nc-Si-embedded mixed-phase Si_1 − *x* − *y*_C_*x*_O_*y*_ seems to provide a promising route for Si-based third-generation photovoltaics.

## Conclusions

In summary, we have performed thermal annealing experiments on Si_1 − *x*_C_*x*_ with various film structures and compositions. As a result, we have found that stoichiometric changes and oxygen incorporation of the matrix Si_1 − *x*_C_*x*_ significantly affects the light absorption properties of mixed-phase Si_1 − *x*_C_*x*_ thin films. This clarifies the strategy towards implementing a light absorber of third-generation photovoltaics: nc-Si-embedded mixed-phase Si_1 − -*x* − *y*_C_*x*_O_*y*_ with pre-existing low-temperature deposition technologies.

## Competing interests

The authors declare that they have no competing interests.

## Authors' contributions

JM performed the experiments and analyses, drew the figures, and wrote the text. SJB directed the analysis, drew the figures, and finalized the manuscript. BO and JCL organized the project, and JCL designed the experiment. All authors read and approved the final manuscript.
